# Formulation preference, tolerability and quality of life assessment following a switch from lopinavir/ritonavir soft gel capsule to tablet in human immunodeficiency virus-infected patients

**DOI:** 10.1186/1742-6405-6-29

**Published:** 2009-12-22

**Authors:** Ighovwerha Ofotokun, Susan K Chuck, Brian Schmotzer, Kelly L O'Neil

**Affiliations:** 1 Department of Medicine, Division of Infectious Diseases, Emory University School of Medicine, 49 Jesse Hill Jr. Drive, Atlanta, GA 30303, USA; 2 Abbott Laboratories, 200 Abbott Park Rd, Abbott Park, IL 60064, USA; 3 Department of Biostatistics, Emory University Rollins School of Public Health, 1518 Clifton Road, Atlanta, Georgia 30322, USA

## Abstract

**Background:**

Lopinavir/ritonavir (LPV/r) tablet compared to the soft gel capsule (SGC) formulation has no oleic acid or sorbitol, has no refrigeration or food-restriction requirements, and has less pharmacokinetic variability. We compared the tolerability, quality of life (QoL), and formulation preference after switching from LPV/r SGC to the tablet formulation.

**Methods:**

In a prospective, single-arm, cohort study-design, 74 human immunodeficiency virus (HIV) infected subjects stable on LPV/r-based therapy were enrolled prior to (n = 25) or 8 weeks (n = 49) after switching from SGC to tablet. Baseline data included clinical laboratory tests, bowel habit survey (BHS) and QoL questionnaire (recalled if enrolled post-switch). Global Condition Improvement (GCI)-score, BHS-score, QoL-score, and formulation preference data were captured at weeks 4 and 12.

**Results:**

At week 12 post-enrollment; the tablet was preferred to the SGC (74% vs. 10%, p < 0.0001). GCI-overall-tolerability score was 2.46 ± 3.30 on a scale of -7 to +7, with 90% admitting to feeling better or about the same. Stool frequency, consistency, volume, and ± blood improved, however the improvement was significant in "consistency" only (p = 0.03). Aggregate Bowel Habit-Profile improved (BHS-score change = -0.227, p = 0.01). Inverse relationship existed between GCI and BHS (slope = -1.2, p = 0.02) at week-4, suggesting that improved overall-tolerability was related to better gastrointestinal (GI)-tolerance. QoL-scores were stable. Mean reductions in total cholesterol of 9.20 mg/dL (p = 0.02), in triglycerides of 33 mg/dL (p = 0.04), and in HDL of 4.50 mg/dL (p = 0.01) unrelated to lipid-lowering therapy, were observed at week 12.

**Conclusions:**

LPV/r-tablet was well tolerated and preferred to the SGC in HIV infected subjects, with stable QoL and appreciable improvement in GI-tolerability. The unexpected changes in lipid profile deserve further evaluation.

## Background

Lopinavir/ritonavir (LPV/r) tablet is now widely used in combination with other antiretroviral agents in place of the soft gel capsule (SGC) in the treatment of HIV-infection. Developed by the melt extrusion technology and approved by the Food and Drug Administration in 2005 [1], LPV/r tablets have several advantages over the SGC. The tablet allows a reduced pill burden (4 tablets/day vs. 6 SGC/day), is storable at room temperature, has no special food requirement, and has comparable bioavailability to the SGC and less plasma concentration variability [2,3]. Because the tablet formulation lacks oleic acid [1], an excipient believed to contribute to gastrointestinal (GI) intolerance in the SGC, it is expected for the tolerability of LPV/r to improve with the tablet formulation.

In this report, the overall tolerability, GI-tolerance profile, fasting lipid profile, quality of life (QoL), formulation preference and satisfaction were prospectively evaluated in a cohort of clinically stable HIV-infected subjects treated with LPV/r based antiretroviral therapy who were switched from the SGC to the tablet formulation. Because of the advantages of the tablet over the SCG, we hypothesized that the covariates evaluated, with the exception of fasting lipid profile and QoL, would improve within 12 weeks of the switch.

## Results

### Patient demographics

Seventy-four clinically stable HIV-infected subjects were enrolled, with 25 subjects (34%) switching from the SGC to the LPV/r tablet formulation at entry and 49 subjects (66%) having already switched to the tablet (within ≤8 weeks of enrollment). No patients withdrew from the study prematurely. The subjects were predominantly African American (74%) and males (82%). Detailed demographic characteristics are summarized in Table 1.

**Table 1:** Subjects' Demographic Data at Study Entry

	Study population (n = 74)
Male sex [No. (%)]	61 (82)
Race	
African American [No. (%)]	55 (74)
White [No. (%)]	17 (23)
Hispanic [No. (%)]	2 (3)
†On LPV/r tablet at entry	
No [No. (%)]	49 (66)
Yes [No. (%)]	25 (34)
On anti-diarrheal drug	
No [No. (%)]	67 (92)
Yes [No. (%)]	6 (8)
On lipid lowering drug	
No [No. (%)]	54 (74)
Yes [No. (%)]	19 (26)
Median age [years (IQR)]	43 (39-47)
Median weight [Kg (IQR)]	80.5 (69.60-88.60)
Median HIV-1 RNA [copies/ml (IQR)]	0.135 (0.05-0.70)
Median CD4 T-cell counts [cell/μl (IQR)]	294 (157-455)

LPV/r, lopinavir/ritonavir; SGC, soft gel capsule; IQR, inter-quartile range.

### Formulation Preference and Satisfaction

At week 4 post-enrollment, a significantly greater proportion of patients preferred LPV/r tablet to SGC formulation (78% vs. 9%, p < 0.0001), and this pattern was maintained at week 12 (74% vs. 10%, p < 0.0001) (Table 2). Medication satisfaction scores for LPV/r tablet were 9.01 ± 2.27 and 8.69 ± 2.25 at week 4 and 12 respectively on a MSS (MSS) scale of 0 to12, where a higher number represents superior outcome.

**Table 2:** Quality of Life, Medication Satisfaction, and Tolerability Scores

Quality of life instruments	Baseline	Change from baseline to week 4		Change from baseline to week 12	
	
	Mean (SD)	Mean (SD)	P-value	Mean (SD)	P-value
*Physical health summary score (PHS)	48.2 (11.5)	0.015 ± 9.00	0.97	0.315 ± 9.20	0.79
*Mental health summary score (MHS)	50.7 (12.0)	0.366 ± 9.07	0.74	0.313 ± 10.01	0.81
*Augmented symptom distress module (ASDM)	26.7 (19.8)	-2.99 ± 16.34	0.14	-2.92 ± 16.48	0.17
*Center for Epidemiology Studies-Depression (CES-D)	14.5 (10.2)	-1.12 ± 8.00	0.25	-0.753 ± 8.47	0.49
		**Mean value at week 4**		**Mean value at week 12**	
		
Medication satisfaction survey (MSS)		9.01 ± 2.27	NA	8.69 ± 2.25	NA
Global Condition improvement (GCI)		2.24 ± 3.05	<0.0001	2.46 ± 3.30	<0.0001
Therapy preference					
Prefer LPV/r tablet [No. (%)]		55 (78%)	<0.0001	46 (74%)	<0.0001
Prefer LPV/r SGC [No. (%)]		6 (9%)		6 (10%)	
No preference [No. (%)]		9 (13%)		10 (16%)	

*These instruments were scored according to the published scoring algorithm [4-6]; SD, standard deviation; SGC, soft gel capsule; LPV/r, lopinavir/ritonavir;

### Formulation Tolerability

The GCI-tolerability scores were 2.24 ± 3.05 at week 4 (p < 0.0001) and 2.46 ± 3.30 at week 12, (p < 0.0001). Scores were on a scale of -7 to +7 where a positive integer is an indication of improved overall tolerability with the tablet as compared to SGC (Table 2). Ninety percent of subjects either "felt better" (45%) or "felt about the same" (45%), with only 5% expressing feeling worse, while 5% did not respond.

### GI-tolerability

Overall change in daily bowel habit pattern was also improved. Statistically significant changes in mean Bowel Habit Score (BHS) score of -0.281 (p = 0.002) and -0.227 (p = 0.01) were observed at week 4 and week 12, respectively. In the subset of subjects reporting changes in bowel movement pattern, improvements were noted in all four parameters assessed. However, only change in stool consistency reached the level of statistical significance (Table 3). A decrease in stool frequency was reported by 18 of 28 subjects at week 4 (64%, p = 0.13), and by 18 of 32 subjects at Week 12 (56%, p = 0.48). Stool consistency improved in 23 of 30 subjects at week 4 (77%, p = 0.004), and in 19 of 27 subjects at week 12 (70%, p = 0.03). A reduction in stool volume occurred in 11 of 16 subjects at week 4 (69%, p = 0.13), and in 8 of 13 subjects at week 12 (62%, p = 0.41). Resolution of blood in stool occurred in 5 of 6 subjects at week 4 (83%, p = 0.10), and in 4 of 6 subjects at week 12 (67%, p = 0.41).

**Table 3:** Bowel Habit Survey

Variables	Baseline to week 4		Baseline to week 12	
	Improvement rate	P-value	Improvement rate	P-value
Decrease in stool frequency among those reporting change	18/28 (64.3%)	0.13	18/32 (56.3%)	0.48
Improved stool consistency among those reporting change	23/30 (76.75%)	0.004	19/27 (70.4%)	0.03
Decrease in stool volume among those reporting change	11/16 (68.8%)	0.13	8/13 (61.5%)	0.41
Resolution of blood in stool among those reporting change	5/6 (83.3%)	0.10	4/6 (66.7%)	0.41
	
	**Baseline to week 4 (n = 70)**		**Baseline to week 12 (n = 62)**	
Overall change in bowel habit score (BHS) [mean (SD)]	-0.281 ± 0.719	0.002	-0.227 ± 0.707	0.01

Self-reported bowel habit score (BHS) was assessed on a scale in which stool consistency was (solid = 1, loose = 3, watery = 5); volume (small = 1, moderate = 3, large = 5), blood (no = 1, yes = 5); Frequency (1 - 5). For example, a subject with baseline responses of: Solid, Moderate, no blood, and frequency of "2" would have a score of: (1 + 3 + 1 + 2)/4 = 1.75 for their baseline summary score. Therefore the scale has a minimum of 1 (best BHS outcome) and a maximum of 5 (worst BHS outcome); SD, standard deviation.

### Impact of GI-tolerability on overall formulation tolerability

To examine whether the change in Global Conditioning Improvement-tolerability score was resultant of the change in BHS-score, a simple linear regression was performed. For every unit reduction in mean BHS-score from baseline to week 4, the GCI-score at week 4 improved by an average of 1.2 points (slope = -1.2, p = 0.02), however, this effect seemed to diminish by week 12 (slope = -0.95, p = 0.11).

### QoL Assessment

The MOS-HIV health survey (Physical Health Summary Score (PHS) and Mental Health Summary Score (MHS)), Augmented Symptom Distress Module (ASDM), and Center for Epidemiology Studies-Depression (CES-D) instruments, which evaluated physical functioning, pain, social functioning, emotional well-being, energy/fatigue, health transition, and overall QoL, showed no differences between the SGC and tablet formulations (Table 2).

### Fasting lipid profile

In a subset of subjects not treated with lipid lowering agents, mean changes of -9.2 mg/dl (n = 44, p = 0.02) in total cholesterol, -33.2 mg/dl (n = 38, p = 0.04) in triglyceride, and -4.5 mg/dl (n = 37, p = 0.01) in HDL were observed at week 12 (Table 4).

**Table 4:** Changes in Fasting Lipid Profile from Baseline to Week 12

		**Baseline**	**Week 12**	**Baseline to week 12**		
	
	**Lipid Lowering Therapy**	**Mean (SD)**	**Mean (SD)**	**Mean Change**	**SD**	**P-value**
	
**Total Cholesterol**	No (n = 44)	183 (36.6)	180 (35.4)	-9.2	23.2	0.02
	Yes (n = 18)	225 (64.3)	221 (55.1)	-2.9	44.3	0.80
	Total population	195 (49.7)	191 (45.1)	-7.3	30.7	0.09
						
**Triglycerides**	No (n = 38)	178 (114)	153 (105)	-33.1	86.3	0.04
	Yes (n = 16)	411 (392)	303 (281)	-81.2	348.3	0.40
	Total population	246 (253)	194 (182)	-47.4	199.9	0.11
						
**HDL**	No (n = 37)	45.3 (11.6)	44.6 (13.7)	-4.5	9.4	0.01
	Yes (n = 17)	36.1 (15.5)	39.1 (17.2)	1.7	9.5	0.49
	Total population	42.4 (13.5)	43.0 (14.8)	-2.5	9.8	0.88
						
**LDL**	No (n = 38)	106 (28.0)	105 (25.9)	-4.2	21.8	0.28
	Yes (n = 16)	127 (54.9)	125 (36.1)	3.6	48.6	0.79
	Total population	112 (38.7)	110 (30.2)	-1.9	31.8	0.69

HDL = high density lipoprotein; LDL = low density lipoprotein; SD = standard deviation

## Discussion

LPV/r is a boosted protease inhibitor (PI) with potent antiviral activity against wild-type and resistant HIV-strains [4,5]. In clinical practice, it is extensively used in treatment naïve and treatment experienced patients, and it is a recommended first-line drug in treatment naïve patients according to the Department of Human and Health Services (DHHS) guidelines [6]. In its original formulation, the SGC, the use of this drug was limited by its high pill burden (6 capsules/day), the need for administration in fed state to optimize bioavailability, refrigeration storage requirement, and gastrointestinal intolerance [3].

The meltrex-engineered LPV/r tablet was introduced to overcome these shortcomings. The melt extrusion technology improves bioavailability of poorly soluble compounds, such as LPV/r, by dissolving the drug in polymer in a solvent-free environment. The drug remains in a state of molecular dispersion as the polymer hardens. This extruded material can then be shaped as tablets, granules, pellets or powder which can be further processed into conventional tablets [2]. The LPV/r tablet thus represents an improvement over the SGC as it is stable at room temperature, has a reduced daily pill count, and has no special food requirement. The tablet formulation has been shown to have comparable pharmacokinetic profile to the SGC formulation with an added advantage of less inter-subject plasma concentration variability [2]. Furthermore, Study M05-730 demonstrated patients' preference for the tablet after switching from the SGC [7].

The results of the current study confirm the overall tolerability of the LPV/r tablet among HIV-infected subjects, its improved GI-tolerability profile over the SGC, and a preference for its use among individuals who underwent a formulation switch. Statistically significant improvements were observed for all measures of medication satisfaction at both the 4 and 12 week evaluation periods. Seventy-eight percent and 74% of subjects preferred the tablet LPV/r to the SGC at weeks 4 and 12, respectively. The Global Condition improvement scale, a validated instrument which ranks medication tolerability in terms of whether subjects felt worse or better with a given drug, indicated an improved overall tolerability following switch to the LPV/r tablet. The week 4 GCI tolerability score was 2.24 ± 3.05 (p < 0.0001), while the week 12 GCI-score was 2.46 ± 3.30 (p < 0.0001) on a scale ranging from -7 to +7. The Medication Satisfaction Survey scores at week 4 of 9 ± 2.27 out of 12 points (75%), and at week 12 of 8.69 ± 2.25 out of 12 points (72%) were consistent with the therapy preference scores and further validate the overall preference of the tablet over the SGC. The preference of the tablet over the SGC is likely due to its reduced pill burden, lack of a food requirement, and its ability to be stored at ambient temperature.

GI-intolerance is commonly associated with PI therapy, including LPV/r SGC [8,9]. It has been speculated that the LPV/r tablet would have less GI side effects than the SGC, because it lacks components that were present in the older formulation (e.g., oleic acid) that may act as a laxative [10]. Indeed, our results indicated an improved trend in all parameters of bowel habits assessed. Among subjects reporting a change in their bowel habit pattern, improvements were noted in stool consistency (less loose), frequency (decreased), volume (decreased) and the presence of blood (reduced). Only stool consistency reached the pre-defined significant α-level of ≤0.05 however. In addition, aggregate change in bowel habit profile, as assessed by change in mean BHS-score, was statistically significantly improved at week 4, and this was maintained through week 12 (-0.281, p = 0.002; and -0.227, p = 0.01, respectively). These changes in GI-tolerability observed with the tablet are consistent with the observed preference of the tablet to the SGC. Furthermore, when interpreted in the context of a cohort of stable patients already tolerant of the SGC, the observed improvement in GI-tolerability is notable. For HIV-infected patient naïve to LPV/r, the tablet formulation might exhibit a superior GI-tolerance profile than has been reported previously for this drug. Corroborative findings of improved GI-tolerability and preference of the tablet over the SGC reported in a number of recent small cohort studies lend additional support to this speculation [10-12]. It should be noted however that difference in GI-tolerance between the SGC and the tablet formulation was not observed in the larger M05-730 [7]. A possible explanation for this difference could be in the design of the two studies. Whereas the current study employed instruments specific for capturing details of GI symptoms, in the M05-730, this information may have been obtained as part of the general adverse event reporting.

Additionally, we observed a linear inverse relationship between overall tolerability of the LPV/r tablet and bowel habit pattern. For every unit reduction in BHS-score from baseline to week 4, the GCI tolerability score at week 4 improves by an average of 1.2 points (slope = -1.2, p = 0.02). Although improvement in overall tolerability with the LPV/r tablet, as assessed by the GCI-score, might be attributable to multiple factors; this correlation between the GCI and BHS scores does suggest that it may, in part, be related to better GI-tolerability with this formulation. However, the strength of this relationship seemed to wane by week 12 (slope = -0.95, p = 0.11).

Because the study population consisted of patients who were clinically stable on a LPV/r-based antiretroviral therapy, we did not expect an appreciable change in QoL. It was therefore reassuring that following the formulation switch, all measures of QoL (MOS-HIV health survey (PHS and MHS), ASDM, and CES-D instruments) assessed in this study were stable. In addition, contrary to our expectation, mean reductions in total cholesterol of 9.20 mg/dL (p = 0.02), in triglycerides of 33 mg/dL (p = 0.04), and in HDL of 4.50 mg/dL (p = 0.01) unrelated to lipid-lowering therapy, were observed at week 12. The reason for this observed lipid lowering effect is unclear and deserves further evaluation in larger cohorts.

Finally, the findings of this study should be interpreted in the context of a pilot, single arm study with a sample size of 74 subjects. Nevertheless, it is reassuring that similar observations regarding the preference and tolerability of the LPV/r tablet over the SGC have been reported in small, unpublished cohorts [10-12]. Our findings are also limited by the fact that for 66% of the subjects, baseline information was recalled (within 8 weeks) since they had already been switched from the SGC to the tablet formulation prior to study entry. Given these limitations, our findings suggest that the LPV/r tablet is well tolerated in HIV-infected subjects, with appreciable improvement in GI-tolerance profile, stable QoL, and a resultant preference of this formulation of LPV/r over the SGC. The unexpected change in plasma lipid profile observed with this formulation deserves further evaluation in a larger comparative study.

## Conclusions

LPV/r-tablet was well tolerated and preferred to the SGC in HIV infected subjects, with stable QoL and appreciable improvement in GI-tolerability. The unexpected changes in lipid profile deserve further evaluation.

## Methods

### Study Population and Design

This was a prospective, single arm, cohort study that enrolled clinically stable HIV-infected subjects, age ≥18 years, receiving LPV/r-based antiretroviral therapy. Subjects were enrolled prior to, or within eight weeks of, the SGC to tablet formulation switch. There were no CD4 T-cell counts or HIV RNA restrictions. Subjects were excluded if they were pregnant, or breastfeeding. All subjects were recruited from the Grady Health System Infectious Diseases Program (IDP) Clinic in Atlanta, and provided written informed consent before undergoing any study procedures. This study was designed according to the ethical guidelines for human studies and approved by the Emory University Institutional Review Board and Grady Health System Research Oversight Committee.

### Intervention

At baseline, demographic and clinical laboratory data including fasting lipid profile, plasma HIV-1 RNA level, and CD4+ T-cell counts were obtained. QoL was assessed by MOS HIV Health Survey, and Augmented Symptom Distress Module (ASDM) questionnaire. As the target population consisted of clinically stable individuals on therapy, severe GI symptoms were not anticipated. Baseline GI-tolerance profile was assessed by measuring changes in bowel habit pattern using the Bowel Habit Survey (BHS), which evaluated the frequency, consistency, volume, and the presence or absence of blood in stool. Subjects' LPV/r SGC were switched to an equivalent dose of the tablet formulation. For subjects who had switched prior to study entry, clinical laboratory tests obtained just prior to the switch were documented as pre-switch laboratory values, and baseline questionnaire information was recalled. During subsequent visits at week 4 and week 12 post-enrollment, QoL questionnaire and BHS were re-administered. In addition, Therapy Preference Questionnaire, Medication Satisfaction Survey (MSS), and Global Condition Improvement (GCI) questionnaire were administered. Clinical laboratory tests (fasting lipid profile, HIV-RNA PCR, and CD4+ T-cell counts) were obtained at the week 12 visit.

### Statistical Analysis

Outcomes of interest were mean changes from baseline or absolute values at week 12 of medication preference and satisfaction assessment, GCI tolerability-scores, GI-tolerability assessed by the BHS-score, QoL-score, and fasting lipid profile. Questionnaires were scored as follows:

*The Medication Satisfaction Survey (MSS) *included a set of questions on how subjects' medications affected their "normal life", made them "feel sick", or made them "feel about their fight against HIV infection". Responses to these questions were ranked on a 5-point Likert scale. The MSS score ranges from 0 - 12 and is a sum of items on the survey. The score was 0 for someone who answered "all of the time" for all questions, and 12 for someone who answered "none of the time" for all of the questions. Higher numbers represent better satisfaction.

#### The Therapy Preference Questionnaire

There was no "scoring" of this questionnaire because it consists of one question that assessed subjects' preference with regard to formulation type. Proportion of subjects that preferred one or the other formulation were calculated and compared.

*The Global Condition Improvement (GCI) *questionnaire assessed medication tolerability in terms of whether subjects felt worse or better after the formulation switch, and to what extent did their condition changed. The GCI was scored by assigning a value of 0 if the response was "about the same" (felt neither worse nor better). If the response to the initial question was "worse", a negative value was assigned based on the response to the follow-up question. If the response to the initial question was "better", a positive value was assigned based on the response to the follow-up question. The final variable has a range of -7 to +7, with higher positive numbers representing better tolerability.

*The MOS-HIV Health Survey *contains 35 items that cover 11 dimensions of health including physical functioning, pain, social functioning, emotional well-being, energy/fatigue, overall QoL, and health transition. Patient responses were scored according to the published scoring algorithm, which ranges from 0 to 100, with higher scores indicating better health and well-being [13,14]. *Mental Health Summary (MHS) and Physical Health Summary (PHS) *scores were calculated from the MOS-HIV scale using a method that transforms the scores to a standardized scale with a mean of 50 and a standard deviation of 10 for that particular population. Mean MHS and PHS scores above or below the population mean indicated better or worse health-related QoL, respectively.

*The Augmented Symptoms Distress Module (ASDM) *is a measurement of the presence of common symptoms related to HIV infection, and the extent to which they cause distress to the patient. The ASDM included 22 items that were scored on a scale from 0 to 4, a higher score reflecting the presence of more symptoms and/or a greater degree of symptom-related distress [14].

*The Center for Epidemiological Studies-Depression (CES-D) *instrument is a depression questionnaire consisting of 20 items with 1-week patient recall [14,15]. Components of the questionnaire include depressed mood, feelings of guilt and worthlessness, feelings of hopelessness, loss of appetite, and sleep disturbance. Possible scores range from 0 to 60 with a higher score indicating more symptoms and lower QoL. In the original version of this scoring system four items were worded in the positive direction to break set tendencies in responses. However, for the purposes of this study these questions were reversed so that all questions represented the same direction of QoL.

*The Bowel Habit Survey (BHS) *assessed stool consistency (solid = 1, loose = 3, watery = 5), volume (small = 1, moderate = 3, large = 5), presence of blood (no = 1, yes = 5), and frequency (1 - 5). For example, a subject with baseline responses of: solid, moderate, no blood, and frequency of "2" would have a score of: (1 + 3 + 1 + 2)/4 = 1.75 for their baseline BHS score. Therefore the scale has a minimum of 1 (best BHS outcome) and a maximum of 5 (worst BHS outcome). Subjects' change in stool consistency, volume, presence of blood, and frequency was compared.

#### Analytic approach

Baseline characteristics were summarized by descriptive statistics. Changes in the various covariates from baseline to week 4 or week 12 were tested using a paired t-test for continuous outcomes. Proportions were tested using a chi-square test. Additionally, the proportion of subjects that had an improvement in stool consistency, volume, presence of blood, and frequency was also compared. A linear regression was also performed to examine the relationship between change in GCI scores and change in BHS scores.

## Competing interests

SKC and KO are employees of Abbott Laboratories.

## Authors' contributions

All authors contributed to interpretation of the data and provided critical review and approval of the manuscript. Additionally, IO designed the study and protocol, conducted the study, and collected data. BS carried out the statistical analyses.
